# Unraveling the Big Sleep: Molecular Aspects of Stem Cell Dormancy and Hibernation

**DOI:** 10.3389/fphys.2021.624950

**Published:** 2021-04-01

**Authors:** Itamar B. Dias, Hjalmar R. Bouma, Robert H. Henning

**Affiliations:** ^1^Department of Clinical Pharmacy and Pharmacology, University Medical Center Groningen, University of Groningen, Groningen, Netherlands; ^2^Department of Internal Medicine, University Medical Center Groningen, University of Groningen, Groningen, Netherlands

**Keywords:** cell cycle, cell dormancy, hibernation, metabolism, torpor, quiescence

## Abstract

Tissue-resident stem cells may enter a dormant state, also known as quiescence, which allows them to withstand metabolic stress and unfavorable conditions. Similarly, hibernating mammals can also enter a state of dormancy used to evade hostile circumstances, such as food shortage and low ambient temperatures. In hibernation, the dormant state of the individual and its cells is commonly known as torpor, and is characterized by metabolic suppression in individual cells. Given that both conditions represent cell survival strategies, we here compare the molecular aspects of cellular quiescence, particularly of well-studied hematopoietic stem cells, and torpor at the cellular level. Critical processes of dormancy are reviewed, including the suppression of the cell cycle, changes in metabolic characteristics, and cellular mechanisms of dealing with damage. Key factors shared by hematopoietic stem cell quiescence and torpor include a reversible activation of factors inhibiting the cell cycle, a shift in metabolism from glucose to fatty acid oxidation, downregulation of mitochondrial activity, key changes in hypoxia-inducible factor one alpha (HIF-1α), mTOR, reversible protein phosphorylation and autophagy, and increased radiation resistance. This similarity is remarkable in view of the difference in cell populations, as stem cell quiescence regards proliferating cells, while torpor mainly involves terminally differentiated cells. A future perspective is provided how to advance our understanding of the crucial pathways that allow stem cells and hibernating animals to engage in their ‘great slumbers.’

## Introduction

The difference between life and death of individual cells or animals depends on their ability to survive, particularly during periods of scarcity. When environmental conditions are unfavorable, or nutrients are scarce, individual cells may enter a dormant state (quiescence). Similarly, some mammals may hibernate to cope with such conditions by suppressing metabolism in a state called torpor. Given that they represent cell survival strategies triggered by external factors, cell quiescence and hibernation both deploy molecular adaptations to survive environmental stress such as low temperature and shortage of nutrients, and even increase their resistance to withstand periods of low oxygen supply. In both cases, phenotypic plasticity is of paramount importance to ensure survival, yet it is undocumented whether the mechanism governing entry or exit from cellular dormancy and torpidity are similar. Here, we summarize mechanisms used in cellular quiescence and mammalian hibernation and use the collective findings to establish their resemblance.

### Cellular Dormancy

Cellular dormancy is the ability to enter a quiescence state (reversible cellular arrest) by withdrawing from the cell cycle and entering the so called G_0_ phase (Nakamura-Ishizu et al., 2014). The cell cycle is divided into four phases: G_1_ phase (interphase), S phase (DNA synthesis), G_2_ phase (interphase), and M phase (mitosis) (Yang and Sheridan, 2014). Cells that overcome the G_1_ checkpoint commit to divide and proceed to the S phase, culminating in cell division. In the early G_1_ phase, cells that are non-proliferating, undivided, senescent (permanent cell cycle arrest) or terminally differentiated, can withdraw from the cell cycle and enter a dormant or quiescent state (G_0_) (Figel and Fenstermaker, 2018). Quiescent cells are characterized by low mobility, low metabolic activity and rare division (Rocheteau et al., 2014). Once quiescent, cells may either re-enter the G_1_ phase in response to growth signals and commit to divide (reversible quiescence) or continue dormancy, which may or may not ultimately lead to senescence, i.e., a state of permanent cell cycle arrest with high metabolic activity and secretion of inflammatory factors (Sabbatinelli et al., 2019). In contrast, non-proliferating cells that are either terminally differentiated or senescent are irreversibly arrested.

In humans, reversible quiescence commonly occurs in many somatic cells including hematopoietic stem cells (Nakamura-Ishizu et al., 2014), muscle stem cells (Chakkalakal et al., 2012), epithelial stem cell (Coloff et al., 2016), neural stem cell (Kalamakis et al., 2019), and hair follicle stem cell (Wang et al., 2016). Although quiescence is a key characteristic of tissue-resident stem cells, which function as a dormant reserve to replenish the tissue loss throughout life, the discovery of highly proliferative stem cells in several tissues has challenged the concept that quiescence is an integral property of all stem cells (Clevers and Watt, 2018). Given the divergent mechanisms governing quiescence in different stem cells, this review will focus on the most extensively and well-characterized tissue-resident stem cells: hematopoietic stem cells (HSCs) (Richmond et al., 2016). Nevertheless, quiescence can also be found in non-stem cells such as endothelial cells (Sabbatinelli et al., 2019) and mature hepatocytes, the latter being considered long-term quiescent cells essential for liver regeneration (Zimmermann, 2004; Berasain and Avila, 2015). Although quiescence is not an inherent property that characterizes stem cells or distinguishes them from non-stem cells (e.g., consider mature hepatocytes), dysregulation and loss of quiescence affects homeostasis of many progenitor cell populations, ultimately leading to stem cell exhaustion, i.e., the depletion of stem cells with impact on health (Orford and Scadden, 2008). Stem cell exhaustion is particularly noticeable in HSCs, due to their multi-lineage capacity of differentiation and self-renewal potential (Pietras et al., 2011). HSCs give rise to progenitor cells that differentiate into all lineages of mature blood cells. However, continuous self-renewal of HSCs is insufficient for lifelong maintenance, as the inevitable accumulation of damage would result in dysfunctional hematopoiesis, leading to diseases such as leukemia (Riether et al., 2015). Hence quiescence is considered an essential feature to prevent HSCs exhaustion. To avoid this potential hazard, HSCs are kept quiescent in a unique microenvironment in the bone marrow. Quiescence is actively maintained in HSCs, in which the microenvironment plays a crucial role to assure their longevity and function. Furthermore, computational modeling of HSCs kinetics infers that human HSCs complete the cell cycle once every 18 years to self-renew and generate progenitor cells. Quiescence thus allows stem cells to prolong their lifespan to maintain critical physiological functions (Hao et al., 2016).

### Animal Dormancy

Dormancy in animals can be subdivided into four subclasses: hibernation (Lee, 2009), diapause (Renfree and Shaw, 2000), estivation (Masaki, 2009; Storey and Storey, 2012), and brumation (McEachern et al., 2015). Hibernation is often described as winter dormancy and is adopted by both warm and cold-blooded vertebrates. Furthermore, hibernation is characterized by alternating periods of low metabolic activity (torpor), and normalization of metabolism and physiology (arousals) (Carey et al., 2003). Diapause refers to a spontaneous interruption of the development, characterized by a reduction of metabolic activity and is mainly observed in insects and a few mammalian species (Renfree and Shaw, 2000; Denlinger, 2002). Estivation occurs in vertebrates and invertebrates and is characterized by reduced metabolic rate and inactivity to avoid desiccation during hot periods with soaring temperatures (Masaki, 2009; Storey and Storey, 2012). Brumation is mostly seen in reptiles and strictly induced by low ambient temperatures. It is characterized by long periods of inactivity with lowered respiration rate, intersected by brief periods of activity required to drink (McEachern et al., 2015). Despite having evolved different forms of dormancy, the end goal of these animals is the same: survival of periods with low energy supply.

Between these forms of animal dormancy, hibernation is the best explored regarding molecular changes, which is why we will almost exclusively focus on hibernation in mammals. Depending on the species and environmental challenges hibernation takes different forms, mostly consisting of seasonal hibernation with multi-day torpor bouts and brief arousals (12–18 h) *versus* daily torpor during which metabolism is suppressed for 6–10 h (Turbill et al., 2011). Likely, similar molecular mechanisms govern both, as some species switch between multi-day and daily torpor (Wilz and Heldmaier, 2000; Dausmann et al., 2004). Hibernation consists of (daily) torpor phases that are characterized by low metabolism, which are interspersed by brief periods of arousal with restoration of metabolic rate to levels of non-hibernating animals. Torpidity is a state in which physical activity, development, growth and metabolism are transitorily and profoundly reduced in response to harsh environmental conditions and to reduce energy dissipation (Heldmaier et al., 2000). In this review, we discriminate between the two different forms of torpidity by using ‘torpor’ and ‘daily torpor.’

During torpor, animals undergo profound physiological, morphological and behavioral changes. For example, body temperature of seasonal hibernators in cold environments sharply declines to as low as 0–4°C, heart rate and respiration decreases by 95%, and renal function is significantly reduced (Carey et al., 2003). Most hibernators synchronize their dormancy with environmental changes, with some animals entering dormancy only after the start of unfavorable conditions (consequential dormancy, such as facultative hibernation in the Syrian hamster), while others have a yearly rhythmicity allowing them to enter in advance of harsh conditions (predictive dormancy, such as seasonal hibernation in ground squirrels) (Lee, 2009; Masaki, 2009; Ruf and Geiser, 2015).

### Prerequisites for Dormancy

Cellular quiescence in HSC is associated with three key changes in cell physiology: (i) cell cycle arrest by inhibition of cyclin-dependent kinases (CDKs) upon an increase in expression of cyclin-dependent kinase inhibitors (CKIs), (ii) lowering of metabolism with a switch from carbohydrate to lipid-based metabolism and (iii) resistance to accumulating cellular damage conferred by differential expression of genes involved in apoptosis, proliferation and oxidative stress.

The exact signals that induce torpidity in mammals are still not known, yet reduction of metabolic rate is at its heart (Heldmaier et al., 2000; Storey and Storey, 2004). Torpor entry is achieved by active suppression of metabolism and by limiting ATP-expensive activities, ultimately leading to a change of physiology in cells, tissues and organs. In torpor, vital functions including respiratory and heart rate strongly decline secondary to the metabolic suppression, while temperature regulation is adjusted to accommodate lower body temperatures (Tsiouris, 2005; Ruf and Geiser, 2015). Moreover, reversible cell cycle arrest has been reported in hypoxic red-eared slider turtles and torpid 13-lined squirrels (Biggar and Storey, 2009; Wu and Storey, 2012a), in concert with the metabolic suppression and shift in energy source from carbohydrates to fatty acids (Storey et al., 2010).

Because of the similarity between the overall regulation of HSC quiescence and torpidity, it is conceivable that some form of reversible quiescence occurs during hibernation and might even be necessary for the induction of hibernation. The interaction between these mechanisms may set the stage for both reversible cellular quiescence and hibernation allowing them to withstand stress conditions and extend their life span. However, it must be underlined that the impact of mechanisms involved, particularly those regulating cell cycle, differ largely between quiescence in stem cells and torpidity. While in stem cells cell cycle regulation drives their proliferation and self-renewal, it is less clear what its role is in terminally differentiated cells, as studied in torpor.

## Cell Cycle Regulation

### General Regulation of the Cell Cycle

**FIGURE 1:**
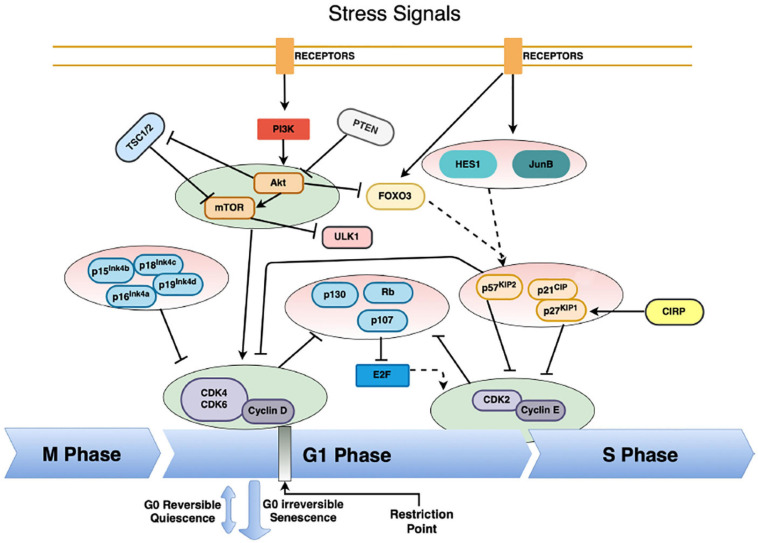
The complex regulation of HSC – and possibly hibernators – cell cycle entry. Cyclin-CDK complexes drive the progression of the cell cycle. Their inhibition forces entry into quiescence from G_1_ into the G_0_ phase of the cell, mainly by the action of cyclin-dependent kinase inhibitors (CKI) from the INK and CIP/KIP family. Various pathways converge on the CKIs, thus instituting quiescence. Low activity of the PI3K/Akt/mTOR pathway is vital to enter quiescence, mainly through the inhibition of cyclin-D-CDK4/6 complexes and suppression of the Rb-E2F pathway. This pathway is massively regulated, primarily through inhibition by the tumor suppressors phosphatase and tensin homolog (PTEN) and tuberous sclerosis proteins 1 and 2 (TSC1/2). Furthermore, the activity of the cyclin D-CDK4/6 complexes is inhibited both by the Ink4 and CIP/KIP CKIs. The Cyclin E-CDK2 complex regulates progression from the G_1_ to the S phase, and it is mainly regulated by the CIP/KIP family of CKIs, as well as by tumor suppressors from the retinoblastoma protein family (Rb, p107, p130). Extrinsic signals activate the transcription factors HES1, JunB and FOXO3a expression, which in turn regulate the transcription of the CIP/KIP family members. Moreover, entry into cellular quiescence is also subject to regulation via the tumor suppressor p53, as a response to cellular damage. Solid arrows indicate direct activation pathways, solid T-lines indicate inhibitory pathways and dashed arrows indicate transcriptional regulation activity.

While numerous intrinsic and extrinsic factors regulate the cell cycle, they generally converge on the cell cycle master regulators, cyclin-dependent kinases, CDKs (Fisher, 1997; Coller et al., 2006; Valcourt et al., 2012; Cheung and Rando, 2013; Lim and Kaldis, 2013). CDK activation requires binding to the proper cyclin regulatory subunit (A, B, D, and E) and together they drive cell cycle progress (Murray, 2004; Garcia and Su, 2008; Figel and Fenstermaker, 2018). Differential gene expression of cyclins during specific cell cycle phases drives cell cycle progression or arrest (Malumbres and Barbacid, 2005; Camins et al., 2013). Cyclin-CDK complex formation is antagonized by CKIs that competitively bind to the catalytic site of the cyclin-CDK complex (Dai and Grant, 2003; Sánchez-Martínez et al., 2015). Two families of CKIs exist, namely INK4 (p16^INK4a^, p15^INK4b^, p18^INK4c^, p19^INK4d^) and CIP/KIP (p21^cip1^, p27^kip1^, p57^kip2^). The balance between CDKs, cyclins and CKIs determines whether a cell will commit to proliferation or maintains cell cycle arrest. The major factors contributing to regulation of the cell cycle with relevancy to quiescence or torpor are depicted in Figure 1.

Mitogen-induced signaling pathways tightly regulate cell growth and proliferation. In the presence of plentiful nutrients, growth factors activate transmembrane receptors, eliciting downstream signaling cascades, including the rat sarcoma oncogene (RAS) (Beauséjour et al., 2003), myelocytomatosis (Myc) (Rahl et al., 2010) and the serine/threonine-protein kinase B (PI3K/Akt) pathways. This is followed by sequential activation of the mitogen-activated protein kinases (MAPKs), which induce the transcription of cyclin D that binds to cyclin-dependent kinases (CDK) 4 and 6, forming activated complexes that initiate the downstream phosphorylation of DNA synthesis associated proteins (Seger and Krebs, 1995; Pimienta and Pascual, 2007; Risal et al., 2016; Seger and Wexler, 2016). The cyclin-D-CDK(4,6) complex phosphorylates and inactivates the tumor suppressor retinoblastoma protein (Rb) and its homologs p107 and p130. Rb inhibition releases its inhibiting of the E2F transcription factor, thus activating E2F-binding to DNA promoter regions (Infante et al., 2008; Kent and Leone, 2019), allowing the transcription of E2F-dependent genes such as cyclin A and cyclin E. These cyclins form a complex with CDK-2, activating its kinase activity. Cyclin(A,E)-CDK-2 complexes further phosphorylate Rb resulting in its complete inactivation (Dyson, 1998; Harbour and Dean, 2000; Bracken et al., 2004). The increase in cyclins and the activated cyclin(D,E)-CDKs(2/4/6) complexes are essential to drive cells from G1 to the S phase and commit the cell to proliferation.

On the other hand, the absence of growth factors reduces the activity of the RAS (MAPKs) and PI3K/Akt pathways, thus leading to the activation of the glycogen synthase kinase 3 beta (GSK3β), which halts the cell cycle by phosphorylation and subsequent degradation of cyclin-D (Dong et al., 2005; Theeuwes et al., 2017; Tessier et al., 2019). Degradation of cyclin D reduces cyclin(D)-CDK(4,6) complex formation followed by an increase in activated Rb, leading to strong suppression of the E2F transcription factor and downstream genes. Cyclin D thus comprises a rate-limiting factor of cell cycle progression through G_1_ (Trimarchi and Lees, 2002; Malumbres and Barbacid, 2009). Quiescent cells display low levels of activators of the cyclin-CDK-Rb-E2F pathway, such as cyclin D, CDK 2, 4, and 6, and high levels of the pathway repressors, including Rb protein and family homologs (p107 and p130) (Harbour and Dean, 2000; Bracken et al., 2004; Peng et al., 2015) and the CKIs p21 (Cheng et al., 2000) and p27 (Coats et al., 1996). However, when conditions turn favorable again, quiescent HSCs increase Cyclin D and E abundance, thus outcompeting CKIs and activating CDK2/4/6 (Murray, 2004; Choi and Anders, 2014; Figel and Fenstermaker, 2018).

### Cell Cycle Regulation in Cell Quiescence

#### Cell Cycle Arrest in Quiescence

Hematopoietic stem cells quiescence primarily results from cell cycle arrest through inhibition of CDKs by an increase in the abundance of CKIs. When conditions for HSCs survival are unfavorable, intrinsic and extrinsic signals upregulate the expression of CKIs, modulating the formation of cyclin-CDK complexes and allowing the formation of the Rb-E2F complex, thus effectively halting the cell cycle. Meanwhile, quiescent HSCs ensure reversibility by upregulation of the chromatin remodeler helix-loop-helix protein 1 (HES1), which promotes transcriptional repression through alteration of chromatin recruiting histone deacetylases (HDACs) (Sang and Coller, 2009), promoting tight packaging of DNA into heterochromatin and downregulation of p21 (Yu et al., 2006; Sang et al., 2008), p27 (Murata et al., 2005), and E2F-dependent proteins (Hartman et al., 2004). Quiescent HSCs deploy additional mechanisms to protect DNA integrity by raising defense mechanisms against excessive oxidative stress to protect cells from accumulating damage and apoptosis. Low levels of reactive oxygen species (ROS) are tolerated by an increased antioxidant defense, including the NADPH-dependent glutathione reductase system (Hosokawa et al., 2006; Jang and Sharkis, 2007), FOXO3a (Miyamoto et al., 2008; Rimmelé et al., 2015), and Sirtuin1 (Ezoe et al., 2008; Matsui et al., 2012). Further, DNA repair systems are enhanced in HSCs, including non-homologous end joining (NHEJ) and p53-mediated DNA damage response (Maynard et al., 2008; Mohrin et al., 2010; Dannenmann et al., 2015). In addition, increased expression of p53 serves to further enhance quiescence in HSCs by upregulation of downstream genes, including p21, Necdin, Gfi-1, BTG2, BAX, and PUMA (Lacorazza et al., 2006; Liu et al., 2009; Asai et al., 2012).

#### Reversing Cell Cycle Arrest in Quiescence

The ability to recommence the cell cycle following reversible arrest is crucial to the functionality of quiescent cells. Upon sufficient extrinsic growth stimulation, MAPKs activate the transcription factor Myc, which promotes the transcription of several cell-cycle promoting genes, including cyclin D, cyclin E, E2F2, and CDK4. Moreover, evidence suggests that the upregulation of Myc (Eilers et al., 1991; Kretzner et al., 1992), E2F (Johnson et al., 1993; Kowalik et al., 1995) and Cyclin E (Blomen and Boonstra, 2007; Fox et al., 2011) alone can drive a cell out of quiescence into cell cycle progression. Nevertheless, downregulation of CKIs activity, p21 (Cheng et al., 2000; Kippin et al., 2005) and p27 (Coats et al., 1996; Rivard et al., 1996), and downregulation of all three retinoblastoma protein family members (Rb, p107, p130) result in quiescence exit and cellular proliferation (Dannenberg et al., 2000; Sage et al., 2003; Viatour et al., 2008). Moreover, the upregulation of cell cycle progression genes leads to a shift in energy metabolism from lipid based oxidation (FAO) back to carbohydrate oxidation (glycolysis). This switch is essential to supply the demand of the ATP-expensive processes to meet the energy demand during proliferation (Valcourt et al., 2012; Takubo et al., 2013). It is of note that quiescent cells may resume cell proliferation only if they express a specific group of genes. One of the genes essential to the reversibility of quiescence is HES1. Hes1 is upregulated in quiescent cells and prevents premature senescence or terminal differentiation in response to specific signals (hypoxia, wnt signaling, Hedgehog pathways) (Baek et al., 2006; Sang et al., 2008). Although the exact mechanism by which Hes1 governs cell quiescence is still unknown, Hes1 can bind to the DNA enhancer site of the CKIs p21, p27, and p57 in HSCs (Yu et al., 2006), expectedly resulting in their inhibition of cyclins, thus arresting the cell cycle.

### Cell Cycle Regulation in Hibernators

#### Cell Cycle Arrest in Torpor

The majority of the pathways involved in stem cell quiescence have also been implicated in hibernators, as torpor phases feature the molecular signature of cell cycle arrest and a reduction of energy-consuming processes such as transcription and translation (Biggar and Storey, 2009; Ruf et al., 2012; Wu and Storey, 2012a; Schwartz et al., 2013; Blanco and Zehr, 2015; Al-attar and Storey, 2020). Modulation of key players in cell cycle arrest due to low nutrient levels is mainly effectuated through the reduction in cellular ATP-consuming processes. A low [ATP]:[AMP] ratio activates the energy-sensing AMP-activated protein kinase (AMPK), initiating a signaling cascade that minimizes energy expenditure. Many genes involved in the cell cycle arrest are upregulated by AMPK dependent phosphorylation, including the transcription factors Hes1, JunB, and FOXO3a. In turn, they induce transcription of CIP/KIP CKIs that strongly inhibit cyclin D and E, which in proliferating cells halts the cell cycle and initiates G_0_ cell arrest (Coller et al., 2006; Li and Bhatia, 2011; Valcourt et al., 2012). In line with these molecular changes, the long term torpor molecular profile matches suppression of cell cycle progression in the liver of thirteen-lined ground squirrel (*Ictidomys tridecemlineatus*) reflected in upregulation of CKIs (p15^*INK*4*b*^ and p21^*CIP*1^) and downregulation of cyclins (D and E) and CDKs2/4/6 (Andrews, 2007). Moreover, the highly proliferative intestinal epithelial cells of thirteen-lined ground squirrel and *Caenorhabditis elegans* embryos (Nystul et al., 2003) halt their mitotic activity during deep torpor because of arrest in the G_2_ phase of the cell cycle (Matthews and Fisher, 1968; Kruman et al., 1988; Kruman, 1992). Yet, similar changes have been observed in terminally differentiated skeletal muscle cells of Brand’s bat (*Myotis brandtii*) (Wu and Storey, 2016) and brown adipose tissue of arctic ground squirrel (*Urocitellus parryii*) (Zhu et al., 2005; Yan et al., 2008). Furthermore, cellular stress such as low temperature, UV radiation and hypoxia, upregulates the expression of the cold-inducible RNA-binding protein (CIRP), which induces the translation of the CKI, p27^*KIP*1^. High activity of CIRP has been reported both in HSCs and during torpor in brain, which suggests that this protein plays a crucial role in inducing cell cycle arrest and in inhibiting proliferation of HSCs at low temperatures (Zhu et al., 2016; Roilo et al., 2018).

#### Reversing Cell Cycle Arrest in Arousal

Changes observed in torpor are basically reversed during interbout arousals. While HES1 constitutes an essential factor to leave cell quiescence in HSC, there is currently limited information about HES1 activity in hibernators, warranting further studies to understand its role in hibernation-induced cellular dormancy.

## Metabolic Characteristics

**FIGURE 2:**
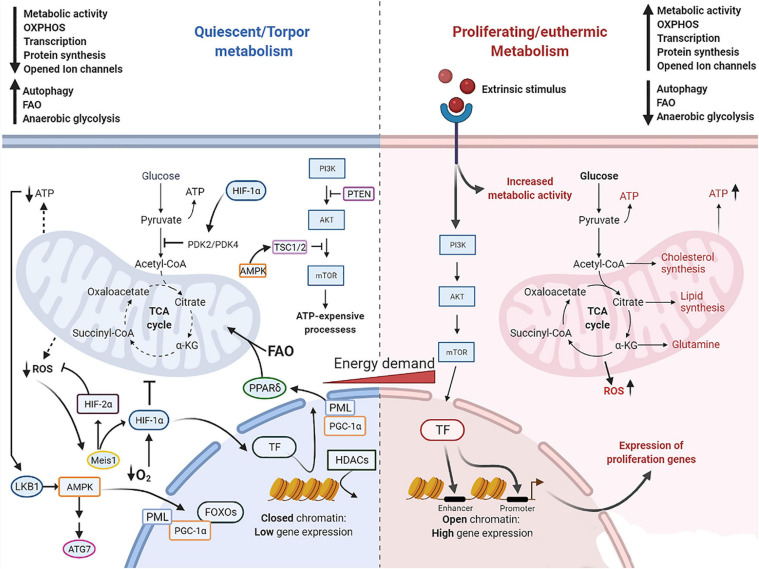
Molecular mechanism governing cell quiescence and torpidity in contrast with cell proliferation and euthermia. Quiescent and torpid cells feature a metabolic shift from glucose fueled OXPHOS to FAO. Low nutrient supply leads to depletion of ATP which is detected by LKB1 that in turn activates AMPK, eliciting a downstream cascade including the activation of TSC1/2 that suppresses PI3K/Akt pathway and downstream ATP-expensive processes. AMPK further shuts down gene expression via activation of HDACs which tightly pack histone-DNA complexes into heterochromatin. Also, AMPK promotes autophagy by activating ULK1/ATG7, lipolysis and fatty acid oxidation via activation of OXPHOS regulator FOXOs proteins and PGC-1α. In addition, PGC-1α, a transcriptional coregulator of PPAR target genes, promotes the upregulation of PPARδ. The PML-PPARδ-FAO further promotes β-oxidation of lipids and the metabolic shift toward FAO. HIF-1α promotes FAO via upregulation of PDK4 and inhibition of the first step in the TCA cycle, and promotes resistance to hypoxia stress and ROS damage. HIF-1α can be activated by its upstream regulator the Meis1 protein, which is activated by the elevation of intracellular ROS, and by low levels of O_2_. Dotted lines represent a restricted generation of molecules pointed at. Solid lines represent activated pathways. T lines indicate the suppression of the pathway. AMPK, AMP-activated protein kinase; ATG7, autophagy related 7; FOXOs, forkhead proteins; HDACs, histone deacetylases; HIF-1α, hypoxia-inducible factor 1α; HIF-2α, hypoxia-inducible factor 2α; LKB1, liver kinase B1; OXPHOS, oxidative phosphorylation; PDK4, pyruvate dehydrogenase kinases 4; PGC-1a, peroxisome proliferator-activated receptor-coactivator 1α; PML, promyelocyte leukemia protein; PML, promyelocyte leukemia protein; PPARs, peroxisome proliferator-activated receptors; PTEN, phosphatase and tensin homolog; TCA, tricarboxylic acid cycle; TF, transcription factors; TSC1/2, tuberous sclerosis proteins 1 and 2.

With their survival depending on an inherent mechanism to maintain adequate metabolic function even during unfavorable environmental conditions, quiescent cells and torpid hibernators undergo a series of interconnected adjustments in metabolism. Key changes in relevant metabolic processes are depicted in Figure 2.

### Metabolic Characteristics of Quiescence

#### Metabolic Switch in Quiescence

One of the remarkable adjustments is the phenotypic switch from glucose fueled mitochondrial OXPHOS to FAO, which involves the differential expression of genes and changes in metabolic processes in response to both intrinsic and extrinsic signals (Lee, 2009; Yao, 2014). Under normal physiological conditions, cellular proliferation requires high levels of energy in the form of ATP, fueled mainly by glucose through mitochondrial OXPHOS of the end product of glycolysis, pyruvate (Yang and Sheridan, 2014). Also, mitochondrial activity, especially oxidative phosphorylation, is the main cellular ROS generator, which can damage macromolecules and organelles, including DNA, proteins and mitochondria (Ito and Ito, 2016). Quiescent cells thus switch from glucose to FAO (β-oxidation), anaerobic glycolysis and autophagy, to secure a minimal energy supply, thereby protecting them from metabolic dysregulation and DNA-damaging ROS production (Valcourt et al., 2012; Ito and Suda, 2014; Yao, 2014). Hypoxia-inducible factor 1α (HIF-1α) is critically involved in this metabolic switch. HIF-1α is a transcription factor expressed in mammalian cells residing under hypoxic conditions, which activates transcription factors and confers post-translational modifications that lower oxygen consumption. HIF-1α promotes anaerobic glycolysis by upregulating glycolytic genes and repressing glucose fueled OXPHOS through transcription of pyruvate dehydrogenase kinases PDK2 and PDK4 (Takubo et al., 2010, 2013). In turn, these kinases inhibit pyruvate dehydrogenase (PDH) (Zhang et al., 2014), which is an essential enzyme to convert glucose derived pyruvate to acetyl-CoA. Inhibition of PDH lowers carbohydrate use by limiting the flow of glycolysis products toward the citric acid cycle and promoting β-oxidation of ketones and fatty acids. At the same time, the lipolytic protein triacylglycerol lipase (PTL), which stimulates lipolysis by breaking down adiposomes (fat droplets), liberates fatty acids for FAO. As HSCs reside in a hypoxic niche in the bone marrow, HIF-1α actively maintains the quiescent state relying on glycolysis and β-oxidation to support low levels of ATP generation. Consequently, inhibition of HIF-1α led to the depletion of mice HSCs, while HIF-1α overexpression induced their quiescence (Takubo et al., 2010). Simsek et al. (2010) showed that HIF-1α expression in HSCs is controlled by the DNA-binding transcription factor myeloid ecotropic viral integration site 1 homolog (Meis1). Further, HSCs upregulate PTL to secure the supply of fatty acids (Liu et al., 2018).

In concert with the metabolic rewiring, adaptations also constitute mechanisms that repress energy-consuming processes by inhibiting gene expression related to cell proliferation, anabolic processes and oxidative phosphorylation through the action of HDACs (Sang et al., 2010). Quiescent cells rapidly reduce their energy expenditure by downregulating ATP-demanding processes, such as DNA replication, macromolecular synthesis, macromolecular turnover and ion pumping activities (Cheung and Rando, 2013; Yao, 2014), which is a requirement for HSC quiescence. Signer et al. showed that chemical induction of protein synthesis leads to the loss of quiescence and promotion of proliferation of mice HCSs (Signer et al., 2014). The switch in energy metabolism back from FAO to glucose fueled OXPHOS is essential to meet the energy demand for differentiation. Indeed, inhibition of mitochondrial respiration blocks differentiation of HSCs (Yu et al., 2013), while inhibition of FAO led to cell proliferation (Ito et al., 2012).

Hypoxia also induces expression of the RNA binding motif protein 3 (RBM3), a critical translation facilitator (Wellmann et al., 2010; Zhu et al., 2019). Loss of RBM3 results in increased damage, mitotic dysfunction and apoptosis (Sureban et al., 2008), reduced neuronal structural plasticity (Peretti et al., 2015) and has been postulated to increase the translational efficacy in HSCs under hypoxic conditions. Other genes that are upregulated in quiescent cells may enhance resistance to apoptosis (NFKB2, MET) (Lin and Karin, 2003; Huh et al., 2004), suppress proliferation (MXI1, TP53, FAT) (Schreiber-Agus et al., 1998; Coller et al., 2006) and protect against accumulating oxidative damage (FOXO, HIF-1α, LKB1, SOD3, PRDX4, EPHX1) (Kops et al., 2002; Shen and Nathan, 2002; Serra et al., 2003; Shackelford and Shaw, 2009; Gurumurthy et al., 2010; Takubo et al., 2010).

#### mTOR in Cell Quiescence

The mammalian target of rapamycin (mTOR) pathway regulates growth and metabolism and embodies a crucial switch from high-expensive energy state (anabolic) to hypometabolism (catabolic). Upstream of mTOR is the highly conserved phosphatidylinositol-3 kinase/protein kinase B (PI3K/Akt) signaling pathway, which is activated by various signals from activated tyrosine kinase receptors. When activated, mTOR drives cell proliferation, growth and survival by activating cyclin-D-CDK4/6 complexes. The Akt/mTOR pathway is regulated by the tumor suppressors phosphatase and tensin homolog (PTEN) and tuberous sclerosis complex (TSC1/2). PTEN and TSC1/2 regulate reversible protein phosphorylation (RPP) of the mTOR pathway determining the “on” or “off” state of many energy-expensive processes. PTEN is considered a major regulator of metabolic reprogramming and has been shown to regulate PDK1, a critical activator of the insulin pathway. Normally, when carbohydrates and glucose are plentiful, insulin signaling activates the PI3K/Akt pathway, which induces glucose uptake and breakdown via glycolysis mechanisms (Rigano et al., 2017; Jansen et al., 2019). In the absence of nutrients, Akt/mTOR is inactivated by the action of PTEN (Yilmaz et al., 2006) and TSC 1/2 (Chen et al., 2008), reducing the energy-intensive processes and inducing catabolism. In a low nutrient environment, levels of glucose are reduced as well as insulin signaling (Martin, 2008; McCain et al., 2013), which reduces PI3K/Akt signaling, activating the forkhead protein (FOXO) family that lowers ROS production to protect from oxidative damage. Quiescent HSCs show the typical changes related to mTOR suppression, including the upregulation of PTEN (Zhang et al., 2006; Kalaitzidis et al., 2012; Porter et al., 2016). In HSCs, activation of the PI3K/Akt/mTOR pathway, reversing the upregulation of PTEN, occurs when cells migrate toward a more oxygen-rich microenvironment, which induces a switch from FAO to glucose fueled OXPHOS and promotes cellular respiration, in turn increasing the levels of ROS and cell cycle progression.

#### Mitochondria in Cell Quiescence

Mitochondrial respiration and production of ROS are strictly regulated by the activity of FOXOs and HIF-1α. Following their activation by growth-repressive signals in the PI3/Akt/mTOR pathway, FOXO proteins repress a large number of mitochondrial genes, inhibiting not only mitochondrial activity, but also biogenesis. Consistent with HSCs’ low dependency on mitochondrial respiration as a source of energy, HSCs are characterized by a low number of mitochondria, which are immature and display underdeveloped cristae with globular morphology (Piccoli et al., 2005). Because of their low metabolic activity, quiescent stem cells produce low levels of ROS (Eckers et al., 2014), yet deploy unique mechanisms protecting them against DNA-damaging ROS (Wanet et al., 2015). The role of Meis1 in counteracting oxidative stress is well established (Kocabas et al., 2012; Unnisa et al., 2012; Papa et al., 2019), by serving as an upstream regulator in response to high ROS levels through activation of HIF-1α and HIF-2α (Simsek et al., 2010; Kocabas et al., 2012; Simonetti et al., 2016). In addition to Meis1, FOXO proteins are essential regulators of oxidative stress and deemed essential to maintain quiescence in long term HSCs. FOXOs inhibit mitochondrial respiratory chain protein expression and transcriptionally activate antioxidant enzymes such as catalases, sestrins, and superoxide dismutase 2 (SOD2). FOXO proteins, notably FOXO3a but also FOXO1, FOXO4, and FOXO6, inhibit mitochondrial gene expression (Tothova et al., 2007; Rimmelé et al., 2015). FOXO3a is highly expressed in HSCs and is also a main transcriptional regulator of antioxidants enzymes (Yalcin et al., 2010; Liang et al., 2016). Conditional deletion of FOXO 1/2/3/4, especially FOXO3a, results in a reduction of the HSC pool (Tothova et al., 2007; Liang et al., 2016; Bigarella et al., 2017). Apart from regulating mitochondrial ROS production and biogenesis, FOXOs also induce the expression of CKIs of the CIP/KIP family (Kops et al., 2002; Camperio et al., 2012), thereby inducing HSC cell cycle arrest. FOXOs are activated by the tumor suppressor liver kinase B1 kinase (LKB1), which also activates AMPK (Shackelford and Shaw, 2009). LKB1 regulates AMPK activity and downstream promotion of ATP production. A relatively high AMP level is indicative of an energy-depleted state and leads to the activation of LKB1, a master kinase that in turn activates the downstream AMPK and 12 other related kinases (Lizcano et al., 2004; Shackelford and Shaw, 2009). Conditional deletion of LKB1 led to loss of quiescence and increased the number of hematopoietic progenitor cells, while depleting HSCs in mice (Gan et al., 2010; Nakada et al., 2010). In addition, LKB1 is an upstream regulator of the peroxisome proliferator-activated receptor-coactivator 1α (PGC-1α), a central regulator of mitochondrial biogenesis and oxidative metabolism, and the deletion of LKB1 in HSCs led to a downregulation of PGC-1α resulting in mitochondrial dysfunction (Gan et al., 2010). On the other hand, the knockout of PGC-1α in early subsets of HSCs showed that hematopoiesis is minimally affected in these cells. Nevertheless, knockout of PGC-1α lead to susceptibility to oxidative stress and modulation of long-term HSC re-population (Basu et al., 2013).

When HSCs commit to proliferation by moving to the high oxygen osteoblastic niche, there is a rapid increase in mitochondrial biogenesis and activity (Jang and Sharkis, 2007; Chen et al., 2008; Valcourt et al., 2012; Nakamura-Ishizu et al., 2014). Proliferating HSCs face high ROS levels and upregulate p38 mitogen-activated protein kinase (MAPK) and mTOR, compatible with the proliferative and differentiation phenotype (Kopp et al., 2005; Parmar et al., 2007). Recent investigation suggests that FOXO3a regulates mitochondrial biogenesis gene transcription, and loss of FOXO3a leads to a dysfunctional metabolic shift and impaired OXPHOS (Rimmelé et al., 2015; Menon and Ghaffari, 2018). However, the mechanism of this regulation remains unknown.

#### Fatty Acid Oxidation in Cell Quiescence

Inhibition of mTOR increases PPARs signaling in HSCs, which has the function of nutrient sensing and transcriptional control of metabolic pathways (Desvergne and Wahli, 1999), especially fatty acid transport and FAO (Braissant et al., 1996; Takahashi et al., 2007). HSC fate and self-renewal decisions are critically dependent on the PML-PPARδ pathway. Promyelocyte leukemia protein (PML) nuclear bodies maintain quiescence in HSC by activating peroxisome proliferator-activated receptors (PPARs), which in turn reprogram cellular metabolism by suppressing the Akt/mTOR pathway. The deletion of PML led to the loss of HSCs quiescence and subsequent exhaustion. Further investigation showed that PPAR signaling and FAO were significantly reduced during HSCs differentiation, while induction of PPARδ by a PML-targeting compound induced quiescence (Ito et al., 2012). The depletion of PML and inhibition of mitochondrial FAO in HSC resulted in symmetric division (two committed HSC daughter cells, i.e., cells with a progressive differentiation toward a particular type of red or white blood cell) both *in vitro* and *in vivo*. Conversely, pharmacological activation of PPARδ increased asymmetric division (one daughter committed, the other for self-renewal) (Ito et al., 2012; Ito and Ito, 2013). Hence, PML and PPARδ activation play an essential role in maintaining the stem cell pool (Lallemand-Breitenbach and de Thé, 2010; Ito and Suda, 2014).

#### Autophagy in Cell Quiescence

Autophagy is a conserved mechanism by which cytoplasmic proteins and organelles are engulfed within autophagosomes and degraded in lysosomes, providing ATP and metabolites. Autophagy is governed by the activation of AMPK in response to nutritional deprivation, leading to phosphorylation of TSC1/2, which in turn inhibits mTORC1 (Yang and Klionsky, 2010). Low levels of mTORC1 activate Unc-51 like autophagy activating kinase, ULK1 (Kim et al., 2008; Mizushima, 2010), which recruits additional proteins to form a complex that promotes autophagosome formation and autophagy. Autophagy related 7 (Atg7) is consistently upregulated in quiescent cells, and deletion of Atg7 in mice decreased the number of HSCs and progenitors cells of various lineages and increased the number of abnormal mitochondria and ROS levels (Mortensen et al., 2011). Furthermore, a mutation in the autophagy gene Atg12 increased levels of ROS, protein synthesis and glucose fueled OXPHOS in mouse HSCs (Vessoni et al., 2012; Revuelta and Matheu, 2017). These results suggest that disruption of autophagy induces a loss of quiescence and a switch back to the proliferating phenotype in HSCs, consistent with the idea that autophagy influences cell fate decisions through metabolic reprogramming of HSC.

### Metabolic Characteristics of Hibernation

#### Metabolic Switch in Hibernation

Hibernators have implemented metabolic adaptations very similar to those found in quiescence. Mammalian hibernators also switch from carbohydrate fueled OXPHOS to lipid-based metabolism (FAO) during torpor (Vermillion et al., 2015). This is reflected in the respiratory quotient (RQ: the quotient of CO_2_ production over O_2_ use), which reflects carbohydrate (RQ ∼ 1.0) *versus* lipid oxidation (RQ ∼ 0.7). During torpor, European hedgehog (*Erinaceus europaeus*) and Arctic ground squirrel show RQ values of 0.7 indicating an exclusive use of FAO. However, during arousals, RQ rises to values > 0.85 suggesting a partial return to glucose fueled OXPHOS metabolism (Tähti, 1978; Buck and Barnes, 2000). Hibernator cells also readily suppress most ATP-consuming processes, including transcription and translation responsible for an estimated 20–30% of cellular energy consumption (Frerichs et al., 1998; Wieser and Krumschnabel, 2001; Armitage et al., 2003). This is thought to be only one of the mechanisms contributing to the metabolic reduction in hibernation, since the metabolic rate reduction during hibernation is much larger (∼83%) (Armitage et al., 2003; Storey and Storey, 2004). Although global transcription inhibition during hibernation is still debated (Carey and Martin, 1996; Hittel D. and Storey, 2002), there is increasing evidence that transcription modulation is tissue-dependent during torpor (Soukri et al., 1996; Van Breukelen and Martin, 2002). For example, thirteen-lined ground squirrels suppress DNA transcription and replication by a twofold decrease in transcriptional initiation, reducing elongation during torpor (Storey, 2003; Tsiouris, 2005; Tessier and Storey, 2014). To avoid energy expenditure by transcription of genes unnecessary in torpor, histone deacetylases (HDACs) silence genes through chromatin remodeling. Protein levels of HDAC1 and HDAC4 are significantly upregulated, and RNA polymerase II activity is downregulated by 57% in thirteen-lined ground squirrels during torpor (Morin and Storey, 2006), suggesting a tight regulation of energy-consuming gene transcription by chromatin remodeling and protein synthesis due to low temperature and low levels of mRNA turnover in hibernation. Given that protein synthesis is even more energetically expensive than transcription, expectedly, most of protein synthesis is actively repressed during torpor. Global mRNA translation is inhibited in torpid golden-mantled ground squirrels (Zhegunov et al., 1988) and Syrian hamsters (Osborne et al., 2004), even though some proteins are tissue-specifically synthesized at low rate during torpor. Conversely, very high rates of protein synthesis and cell proliferation are observed shortly after interbout arousal compared to squirrels that had been active for 1–2 days after hibernation, suggesting an initial compensatory mechanism to replenish proteins lost during torpor (Zhegunov et al., 1988).

Although the mechanistic control of the switch from glucose to fat combustion during hibernation has not been fully elucidated, it seems to be similar to mechanisms observed in starvation (Pilegaard et al., 2003), diabetes (Wu et al., 1999; Kim et al., 2006), and caloric restriction (Lee C. K. et al., 2002). Animals tested in these conditions show upregulation of the transcriptional targets of HIF-1α, including PDK4, PTL, and repression of PDH. However, HIF-1α was not investigated in these studies. Similarly, hibernating ground squirrels showed upregulation of PDK4 and PTL in heart, skeletal muscle and white adipose tissue (Andrews et al., 1998; Buck et al., 2002; Brauch et al., 2005). PTL shows high lipolytic activity at low ambient temperatures as an intrinsic feature of the protein across all mammal lineages. Yet, during torpor, PTL is further upregulated, which is an unique feature observed only in hibernators (Squire et al., 2004). While regulation of PDK4 and PTL only suggests involvement of HIF-1α, direct evidence for its upregulation in torpor comes from two studies. Maistrovski et al. (2012) found increased HIF-1α protein levels in skeletal muscle of 13-lined squirrels and little brown bat (*Myotis lucifugus*), and in liver of little brown bat during hibernation. Previously, HIF-1α protein levels were shown to increase by 60–70% in brown adipose tissue in 13-lined squirrels (Morin and Storey, 2005). Moreover, a recent study using fasting-induced daily torpor in B6N and B6J mice showed that the promoters of the HIF-1α signaling pathway are highly activated during torpidity (Sunagawa et al., 2018). On the other hand, some species such as Arctic ground squirrels (AGS) show significantly higher levels of HIF-1α during late-arousal and euthermic conditions compared to torpor (Ma et al., 2005). These contrasting findings could be due to species differences and/or differences in timing of sample collection. For example, normalization of oxygen consumption rate (OCR) in the brain upon arousal differs greatly among species, taking ∼60 min in Horseshoe bats (*Rhinolophus ferrumequinum*) (Lee M. et al., 2002), while taking ∼4 h in AGS (Zhu et al., 2005). Since the expression and activity of HIF-1α is also regulated by oxygen-independent mechanisms such as ROS levels, mechanical stress, and growth factors (Chun et al., 2002), HIF-1α expression during hibernation might be tissue-specific with differential expression associated with species differences.

In addition to HIF-1α, the hypoxia related RBM3 provides cytoprotection in hibernators by maintaining protein homeostasis under low metabolic conditions (Fedorov et al., 2009). Moreover, several of the genes offering protection in quiescent cell are reported upregulated in hibernating 13-lined ground squirrels, including FOXOs, HIF-1α, SOD3, p21, and p27, as discussed above.

#### mTOR in Hibernation

Hibernation also features changes in expression of the negative regulators of mTOR, i.e., PTEN and TSC1/2. PTEN levels are significantly elevated (1.4 fold) in late torpor in skeletal muscle of thirteen-lined squirrels compared to summer euthermic controls (Wu et al., 2013a, 2015). Activation and inhibition of the Akt/mTOR pathway are paramount for cell cycle arrest during torpor and proliferation during arousal (Huang and Manning, 2008; Wu and Storey, 2012b). Moreover, hibernators re-activate the mTOR pathway upon arousal by supporting the switch from FAO to glucose fueled OXPHOS, increasing oxygen consumption and mitochondrial biogenesis (Wilson et al., 2008; Foudi et al., 2009).

#### Mitochondria in Hibernation

Hibernating thirteen-lined squirrels reduce their O_2_ consumption by 98% from basal levels and increase it by 300% during arousals (Boyer and Barnes, 1999). In addition, an increase in mitochondrial ROS production also activates HIF-1α via the oxidative stress-sensitive transcription factor nuclear factor erythroid 2-related factor 2 (NRF2) (Hawkins et al., 2016; Lacher et al., 2018), which inhibits mitochondrial respiration and sequentially activates LKB1/AMPK (Simsek et al., 2010). However, the underlying mechanism of these molecular processes are not fully understood (Hwang et al., 2014; Li et al., 2015). Recent reports showed a significant upregulation of HIF-1α in the heart and skeletal muscle tissue of thirteen-lined squirrels during hibernation, suggesting that it confers protection against mitochondrial hyperpolarization as a possible mechanism against cellular stress (Maistrovski et al., 2012; Wu et al., 2013b). Mitochondrial hyperpolarization results from the disruption of their electrochemical gradient by the blockade of ATP-synthase, which may ultimately lead to Fas-induced apoptosis (Gergely et al., 2002; Perl et al., 2004). Recently, Ou et al. (2018) reported that exposure of human induced pluripotent stem cell-derived neurons (iPSC-neurons) to low temperature (4°C) produced mitochondrial hyperpolarization and accumulation of ROS, while mitochondrial from iPSC-neurons from thirteen-lined squirrels were depolarized and produced significant less ROS (Ou et al., 2018). High levels of ROS also induced accumulation of HIF-1α in brown adipose tissue (BAT), while HIF-1α knockdown in mice BAT led to reduced levels of glucose consumption, lactate export and glycolytic capacity (Choudhry and Harris, 2018). This suggests that glycolysis is dependent on HIF-1α regulation under hypoxic conditions, which maximizes metabolism in BAT.

Mitochondrial numbers and activity are differentially regulated between torpor and interbout arousal (Martin et al., 1999; Staples, 2014). Mitochondrial activity during hibernation of the 13-lined ground squirrel is tissue-specifically regulated and significantly increases in BAT and brain cortex (Ballinger et al., 2016), while mitochondria number was reported unchanged in ground squirrel liver (Brown et al., 2012), skeletal muscle (Hittel D. S. and Storey, 2002), and heart muscle (Staples and Brown, 2008) during hibernation. Yet, mitochondrial respiration exhibited no apparent suppression in heart muscle, moderate suppression in skeletal muscle and significant suppression in liver. Suppression of the uptake, transport or synthesis of specific substrates of OXPHOS may be a possible mechanism conferring suppression of mitochondrial respiration in liver cells (Brown et al., 2012). This possibility is in line with the decreased succinate dehydrogenase levels during hibernation reported previously (Gehnrich and Aprille, 1988; Cho, 2018).

While LKB1 is considered a master regulator of cellular metabolism in quiescent cells by inhibiting mitochondrial function and biogenesis through activation of FOXO and PGC-1α proteins, its relevance in torpor is still unknown. Nevertheless, in thirteen-lined ground squirrels, AMPK levels in white adipose tissue (WAT) were three times higher than those of summer animals (Horman et al., 2005). The activity of AMPK and LKB1 might be regulated by sex hormones, as dihydrotestosterone (DHT) inhibits AMPK activation, while androgens and estrogens inhibit LKB1 activation (McInnes et al., 2012) and declines in steroid hormone production seem to be a precondition for males to enter hibernation. As such, high levels of testosterone inhibit entrance into torpor in hamster (Hall and Goldman, 1980), hedgehog (Webb and Ellison, 1998), and Belding’s ground squirrel (Lee et al., 1990; Boonstra et al., 2001). Also, transcription of mtDNA and mitochondrial proteins such as PGC-1α, uncoupling proteins (UCP1, UCP3) and AMPK is 4-fold higher in BAT than in other tissues during hibernation (Boyer et al., 1998; Xu et al., 2013; Ballinger et al., 2016). PGC-1α is a central regulator of mitochondrial biogenesis and respiration and it induces the transcription of nuclear respiratory factors (NRF1 and NRF2) that activate the replication of mtDNA.

#### Fatty Acid Oxidation in Hibernation

The crucial role of PML in HSCs quiescence (Ishida, 2009; Ito et al., 2012) would suggest that a similar mechanism is present during mammalian torpor (Lee et al., 2007). However, to the best of our knowledge, there are no reports about the specific role of PML during hibernation, although increasing evidence suggests downstream PPARs to constitute the master transcriptional regulators of changes in lipid metabolism. For example, both protein and mRNA of PPARα were upregulated in WAT, heart, kidney and liver of six species of hibernating bats (Han et al., 2015), jerboa (*Jaculus orientalis*) (Kabine et al., 2004) and 13-lined squirrels (Eddy et al., 2005). PPARα not only induces the activation of genes involved in lipid metabolism, but also induces the expression of uncoupling proteins (UCPs), which pump protons back into the mitochondrial matrix generating heat without synthesizing ATP. The high uncoupled thermogenesis activity of mitochondrial respiration in BAT plays an essential role in thermoregulation and contributes significantly to the rewarming of the organism during arousal from hibernation. Further, PML was also reported to be highly active in thirteen-lined ground squirrels brain during torpor and is associated with massive SUMOylation and increased tolerance to brain ischemia (Lee et al., 2007; Lee and Hallenbeck, 2013).

#### Autophagy in Hibernation

Autophagy is poorly examined in hibernators. In the heart of hibernating Syrian hamster, autophagy seems already initiated during (late) torpor and executed during early arousal, reflected by the gradual increase in active autophagosomes during torpor followed by a peak at early arousal, returning to normal late in arousal (Wiersma et al., 2018). This might be due to the build-up of damaged or misfolded proteins being gradually formed during torpor, since there is an accumulation of cells in the G_2_ phase during torpor (Matthews and Fisher, 1968; Kruman et al., 1988). While autophagy is an essential mechanism to sustain quiescence in HSC, its role in maintaining cell dormancy in hibernators is poorly understood. More research into autophagy in hibernators may unclose relevant knowledge on the preservation of life under stress conditions.

## Radiation Resistance

Under normal physiological conditions, the body has stem cells in all phases of the cell cycle (Lyle and Moore, 2011). Radiation generates high levels of ROS, which increases cellular stress and causes irreversible cellular damage leading to senescence and apoptosis. Particular stem cells are resistant to radiation: cancer stem cells (CSC). Not surprisingly, HIF-1α is significantly upregulated in cancerous cells due to the hypoxic environment created by the rapidly proliferating cells (Majmundar et al., 2010). HIF-1α regulates the switch from glucose to fatty acid combustion, a characteristic of quiescent cells, indicating that HIF-1α might play an important role in conferring radiation resistance in dormant cells. Other mechanisms, including the reduction in histone acetylation because of increased activity of HDACs, lead to a tighter packing of DNA into heterochromatin, which plays a significant role in the radiation resistance of quiescent cells (Diehn and Clarke, 2006). Quiescent cells, especially human stem cells, significantly upregulate antioxidant gene expression, creating an environment where the cells can resist ROS production by radiation (Oberley et al., 1995). In cancer cells, this appears under the control of a particular gene from the FOXO family, FOXM1. FOXM1 downregulation in quiescent cells elevated expression of antioxidant genes such as manganese superoxide dismutase (MnSOD), catalase (CAT), and peroxiredoxin (PRDX3) (Eckers et al., 2014). Radiotherapy is usually administered when treating cancer cells, but quiescent CSCs are resistant to this therapy designed to eliminate proliferating cells (Luk et al., 1986). Knockdown of FOXM1 increases sensitivity to radiation therapy in quiescent and cancer stem cells. The amount of therapeutic radiation is still restricted by its toxicity to normal tissue. For example, multiple metastases cannot be treated without exceeding the tolerance of the healthy organ nearby. By putting specific tissues or organs of patients into dormancy, cells may potentially tolerate higher doses of radiation (Cerri et al., 2016).

Studies conducted in hibernators have shown that torpor limits radiation-induced DNA damage in squirrels, hamsters, and mice (Ghosh et al., 2017; Tinganelli et al., 2019), which has awakened the interest of its utility in cancer therapies and long-haul space missions. The mechanisms that mediate radioprotection during torpor are not known. Likely, they parallel those found in quiescent cells, as torpid animals upregulate antioxidant genes and activate HDACs. Recent studies indicate that radiation resistance in torpor may also relate to the hypothermia, as cell cooling limits DNA damage and leads to a different dynamics in DNA damage repair (Baird et al., 2011).

## Shared Molecular Mechanisms of Cellular Quiescence and Animal Dormancy

### Main Mechanisms Involved

Quiescent (hematopoietic stem) cells and torpor share many similarities (Table 1). First, these include activation of molecular mechanisms that stall the cell cycle of proliferating cells including a major overlap in regulation of essential cell cycle genes and proteins associated with the maintenance of quiescence in HSCs and torpidity during hibernation. In both cases, the entry into dormancy is associated with differential gene expression of proteins that includes cyclins, CDKs and CKIs. Yet the molecular mechanism conferring cellular quiescence in stem cells are described in great detail, whereas this is not the case for hibernators. Moreover, it is evident that induction of HES1 during stem cell quiescence is a prerequisite to enable reversal from cell arrest. However, there is a marked paucity of data on Hes1 activity in hibernators, warranting further studies to understand its role in torpor. HSC may provide a blueprint to disclose mechanisms used by hibernators that govern activation of the molecular machinery of cell cycle arrest in response to environmental changes, even though hibernator cells largely represent terminally differentiated cells.

**TABLE 1:** Comparison of key events driving quiescence in cells to those found in torpid cells.

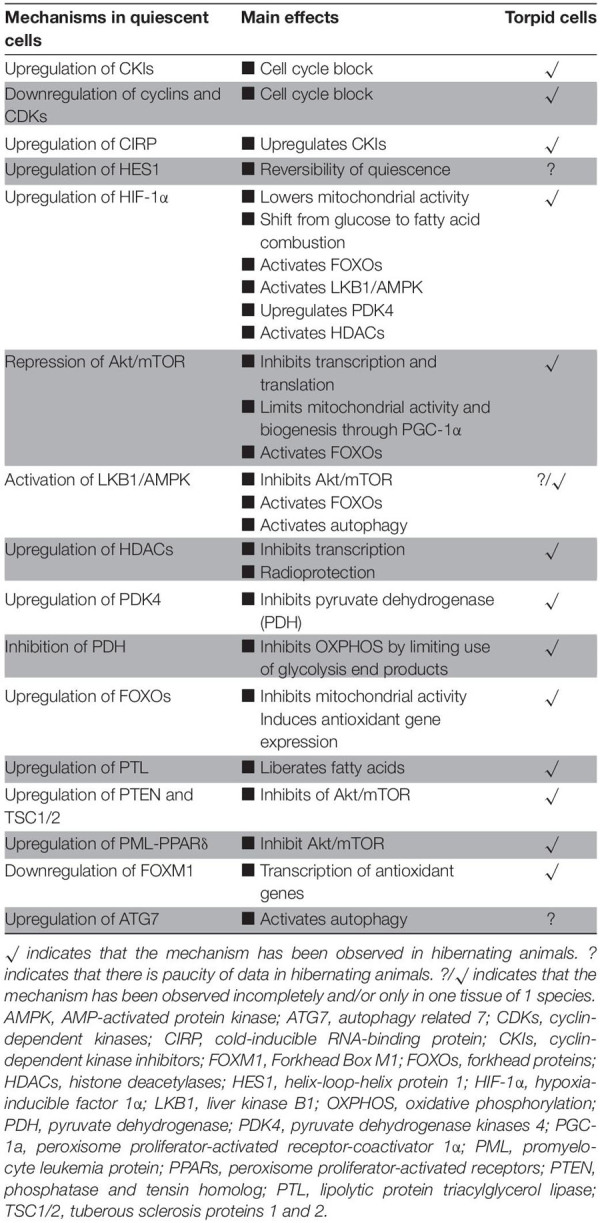

Secondly, cell quiescence and torpor share a similar metabolic rewiring. Both in quiescent and torpid cells, energy conservation is brought about by the reduction in metabolic rate and the switch from glucose fueled OXPHOS to FAO as the primary mechanism to supply ATP, supported by the radical suppression of anabolic processes, such as DNA replication, transcription and protein synthesis. HIF-1α coordinates the cellular adaptation to restore the balance between oxygen supply and metabolic demand, leading to a reduction in the consumption of oxygen. Under ATP-deprived conditions resulting from nutrient deprivation or hypoxia, activation of the energy-sensing liver kinase B1 (LKB1) and the downstream AMP-activated protein kinase (AMPK) precede the upregulation of HIF-1α (Hudson et al., 2002; Lee et al., 2003). Moreover, the activation of LKB1 and AMPK stimulates autophagy through phosphorylation of ULK1 and inhibits the mTOR pathway (Hudson et al., 2002; Li et al., 2015; Mohammad et al., 2019). The Akt/mTOR pathway regulates many energy-expensive processes and is inhibited in quiescence and hibernation by the action of PTEN and TSC1/2 coupled with downstream RPP signaling. High levels of PTEN and TSCs both in HSCs and during hibernation suggest that these proteins are essential to maintain quiescence and torpor. Several of these pathways need further study in hibernators to define their contribution to metabolic suppression, including HIF-1α and autophagy. Further, the PML-PPARδ-FAO pathway appears to play a vital role in the maintenance of HSC quiescence and plays a critical role in its cell fate and self-renewal decisions. However, very little is known about the function and activity of PML during hibernation. Also, quiescent cells and hibernators share the upregulation of cell protective, anti-apoptotic pathways, suggesting that a similar mechanisms is activated during quiescence and mammalian torpor (Heldmaier et al., 2000; Hefler et al., 2015; Zhang et al., 2016).

Thirdly, both quiescent cells and hibernators share radiation resistance, which seems conferred by the combination of upregulation of antioxidant defense and heterochromatin formation. In addition to the antioxidant environment, HIF-1α recruits HDACs to tightly pack the DNA into heterochromatin resulting in resistance to radiation. FOXM1 appears to be a master regulator of antioxidants, and its downregulation resulted in lower radiation sensitivity of cancer stem cells. While strict control over oxidation is crucial for hibernators to survive torpor/arousal switches, there is no literature of the activity and function of FOXM1 in hibernators available. Consequently, mechanisms of radiation resistance in both stem cells and torpor are still ill-defined. At present, it is unclear whether radiation resistance merely exists as a bystander effect of metabolic suppression and antioxidant defense, including a tighter packaging of DNA, or whether it is conferred by specific mechanisms; a question not so easily addressed.

As outlined, the three phenomena discussed above show substantial crosstalk, with the one activating or promoting the other. Although processes are quite similar in HSC and torpor, there may be a clear distinction in their order. For instance, in HSC the regulation of cell cycle and metabolism seems tightly integrated, as interventions in both drive cells out of quiescence. Whether this is true for hibernators is unclear. Possibly, the inhibition of the cell cycle of the differentiated cells in hibernators is merely a consequence of strong metabolic suppression, including the inhibition of DNA synthesis and transcription. To explore differences in orchestration, an accurate delineation of the critical factors and their sequence of events during entry into torpor is warranted.

### Reversible Protein Phosphorylation

Reversible protein phosphorylation (RPP) is a crucial post-translational modifier of proteins and regulator of cell homeostasis. In HSCs and hibernators, RPP is especially important to support the exit from and re-entering of the cell cycle without spending much energy on anabolic processes. While the LKB1-AMPK route plays a crucial role in inactivating many anabolic processes such as protein synthesis, RPP signaling seems of crucial importance to inactivate ion channels. The preponderance of cellular processes that expend energy is directly or indirectly affiliated with membranes preserving concentration gradients. Thus, suppression of membrane-associated (ion channels) proteins by RPP has a profound impact on metabolic reduction and ATP turnover reduction. For example, phosphorylation of sodium-potassium pump (Na^+^/K^+^-ATPase) led to a decrease in activity by up to 60% in golden-mantled squirrels (Storey and Storey, 2004). Therefore, RPP could be responsible for not only maintaining lipid-based metabolism but also to initiate the metabolic repression in hibernation preparation through AMPK signaling.

### Future Directions

The large overlap between quiescent (hematopoietic stem) cells and hibernator adaptations may have some future implications. First, studies on (stem) cell quiescence have identified a number of crucial genes/pathways, of which the relevance in hibernation has been insufficiently explored, in particular LKB1, HES1, HIF-1α, and PML. One study explored the effects of LKB1 knockout in *Caenorhabditis elegans* (Narbonne and Roy, 2009). Interestingly, the LKB1 knockout worms entered Dauer state, a survival mechanism that arrests feeding while retaining activity, motility and acquiring stress-resistance, but rapidly consumed their stored energy leading to failure of vital organs and death. Although *C. elegans* is not a true hibernator nor a mammal, the phenotype is similar to LKB1 knockout in HSC. LKB1 might thus also be an essential protein in hibernators under energetically unfavorable conditions to maintain energy and oxidative stress homeostasis. PML-PPARδ regulation of FAO is essential to maintain HSC quiescence and acts as a negative regulator of Akt/mTOR, a crucial element in the metabolic shift. In hibernators, there is only a single study reporting increased PML-PPARδ activity in thirteen-lined squirrel. Given its critical role in FAO and metabolic reprogramming, PML is an interesting target to address in hibernation. Given the activation of the machinery governing cell cycle block during torpidity, it is of great importance to study HES-1, the factor that retains the option for stem cells to re-enter cell cycle, in particular in relation to arousals. Finally, although upregulation of HIF-1α has been documented in hibernators, details of its regulation and effects need a deeper understanding.

Nevertheless, exploring the relevance of these and other proteins in hibernation is not a trivial task. The main limitation to infer causality in factors contributing to hibernation, is the absence of (conditional) knock-out models in true hibernators. Generation of such models would require genetic modification of hibernator blastocyst stem cells to introduce LoxP sites into genes of interest and generation of Cre expressing lines, preferable harboring inducible promotors, like the tamoxifen-induced CRE-ERT2 system. Such developments may be accelerated by the recent advancements of CRISPR/Cas9 technology, but will still require considerable effort and funding, and specialized knowledge. An alternative approach may be to use inducible knockouts of genes in the house mouse (*Mus musculus*), a species long known to be capable of serial daily torpor (Hudson and Scott, 1979). Conditional knockout mouse lines of a number of factors discussed are readily available. Moreover, the present variety of Cre mouse lines and the superior toolbox to introduce LoxP sites, signify that making the appropriate knockout in mouse is far easier compared to true hibernators. However, it is unclear to date to what extent the molecular mechanisms of mouse daily torpor resemble those of torpor found in true hibernators. The first step should therefore consist of exploring the molecular footprint of cell cycle arrest and metabolic rewiring in mouse torpor. A third option to explore specific genes might be the use of induced pluripotent stem cells (iPSCs) from hibernators (Takahashi and Yamanaka, 2006). These iPSCs can be differentiated into any cell type to study molecular biology *in vitro*. Also, if the selected cell type does not depend on a heterotypic, complex environment, but represents an autonomous cell, it can be further maturated into engineered 3D tissue or organoids to mimic physiological behavior. A recent study showed that iPSC-derived neurons from thirteen-lined squirrel behaved differently than human neurons with higher resistance to cold (Ou et al., 2018). Knocking out genes in iPSC-derived hibernator cells may at least explore the important question whether some of the mechanism present in HSC quiescence may be induced in a cell autonomous way by starvation or hypoxia.

Most cells in a mammal, whether or not a hibernator, are capable of quiescence. The similarity between molecular mechanisms conferring quiescence in HSC and hibernation may signify that there is a unifying (epi)genetic-metabolic program governing the both states. If true, it may mean that all non-hibernator species could be capable of hibernation, provided that cellular quiescence is induced in high energy consuming organs.

## Author Contributions

All authors listed have made a substantial, direct and intellectual contribution to the work, and approved it for publication.

## Conflict of Interest

The authors declare that the research was conducted in the absence of any commercial or financial relationships that could be construed as a potential conflict of interest.
